# Involvement of ubiquitination in Alzheimer’s disease

**DOI:** 10.3389/fneur.2024.1459678

**Published:** 2024-09-05

**Authors:** Nan Lin, Xi-Yan Gao, Xiao Li, Wen-Ming Chu

**Affiliations:** ^1^College of Acupuncture and Tuina of Henan University of Chinese Medicine, Zhengzhou, Henan, China; ^2^The Third Affiliated Hospital of Henan University of Chinese Medicine, Zhengzhou, Henan, China

**Keywords:** Alzheimer’s disease, ubiquitination, protein degradation, Tua, APP, DMT1, AMPARs

## Abstract

The hallmark pathological features of Alzheimer’s disease (AD) consist of senile plaques, which are formed by extracellular β-amyloid (Aβ) deposition, and neurofibrillary tangles, which are formed by the hyperphosphorylation of intra-neuronal tau proteins. With the increase in clinical studies, the *in vivo* imbalance of iron homeostasis and the dysfunction of synaptic plasticity have been confirmed to be involved in AD pathogenesis. All of these mechanisms are constituted by the abnormal accumulation of misfolded or conformationally altered protein aggregates, which in turn drive AD progression. Proteostatic imbalance has emerged as a key mechanism in the pathogenesis of AD. Ubiquitination modification is a major pathway for maintaining protein homeostasis, and protein degradation is primarily carried out by the ubiquitin-proteasome system (UPS). In this review, we provide an overview of the ubiquitination modification processes and related protein ubiquitination degradation pathways in AD, focusing on the microtubule-associated protein Tau, amyloid precursor protein (APP), divalent metal transporter protein 1 (DMT1), and α-amino-3-hyroxy-5-methyl-4-isoxazole propionic acid (AMPA) receptors. We also discuss recent advances in ubiquitination-based targeted therapy for AD, with the aim of contributing new ideas to the development of novel therapeutic interventions for AD.

## 1 Introduction

AD is a progressive neurodegenerative disorder with clinical manifestations of memory loss and cognitive dysfunction. It is the most common form of dementia and with a prevalence, which severely affects patients’ quality of life (1). Recent data from Scheltens et al. in 2021 suggests that the prevalence of dementia will double in Europe and triple globally by 2050. this estimation will be three times higher based on the biological (rather than clinical) definition of AD (2). Understanding the pathogenesis of AD remains a challenge, with the formation of senile plaques due to extracellular β-amyloid (Aβ) deposition and neurofibrillary tangles resulting from hyperphosphorylation of intra-neuronal tau proteins being key hallmarks (3). The misfolding of proteins leads to the aggregation of Aβ and tau into toxic fibrillar structures, impairing their normal functionality (4). Protein homeostasis is maintained through the critical pathway of ubiquitination, with the ubiquitin-proteasome system (UPS) responsible for degrading 80–90% of proteins in the nucleus and cytoplasm. This system is vital for the timely degradation of short-lived, damaged, and misfolded proteins (5). In addition, it has been found that both iron death and synaptic dysfunction are closely associated with the development of AD (6, 7). Thus, the Ferroptosis pathway plays a key role in AD progression, as well as ubiquitination modifications of molecular proteins associated with synaptic plasticity. Notably, ubiquitination modifications of iron-related pathways and synaptic plasticity molecules are implicated in AD advancement. This paper aims to explore the intricate mechanisms of ubiquitination modification in Alzheimer’s disease, offering valuable insights into potential targets for future clinical interventions in AD treatment.

## 2 Ubiquitination

**Figure 1:**
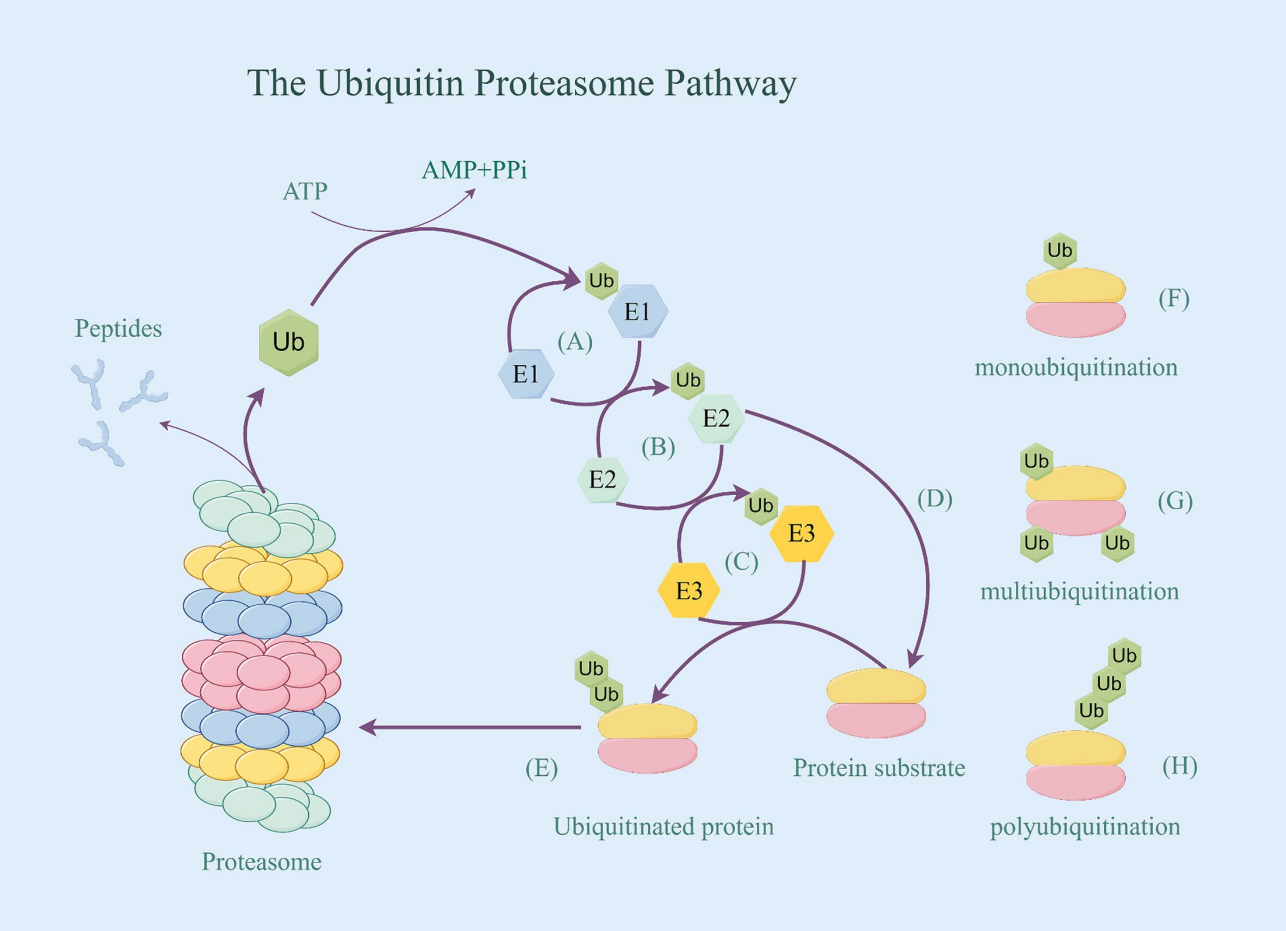
The ubiquitin proteasome pathway. **(A)** Activation: E1’s structural cysteine (Cys) residue and the lysine (Lys) residue at the C-terminus of ubiquitin in the presence of ATP energy bond together to form a high-energy thioester, thereby activating ubiquitin. **(B)** Binding: The activated Ub is then transferred to E2, binding to the Cys residue of E2 through a thioester bond. **(C)** Ligations: E3 transfer the activated ubiquitin to the target protein. **(D)** Activated ubiquitin is attached directly to the protein substrate via the E2. **(E)** Proteins tagged with ubiquitin be identified by the proteasome, thereby being degraded. **(F)** Target protein’s monoubiquitination. **(G)** Target protein’s multiubiquitination. **(H)** Target protein’s polyubiquitination.

Ubiquitination is a vital post-translational modification mediated by ubiquitin (Ub), it is essential for cell division, differentiation, protein quality control, gene expression, DNA repair, protein transport, and signal transduction (8). This intricate process of transferring Ub to a substrate protein by three enzymes undergoes a cascade reaction, which consists of a three-step reaction: a. Activation: Ubiquitin-activating enzymes (E1s) are the initial enzymes necessary for the binding of ubiquitin to substrate proteins. These enzymes, devoid of effects on the specificity of target proteins, form a high-energy thioester by facilitating bond between their structural cysteine (Cys) residue and the lysine (Lys) residue at the C-terminus of ubiquitin in the presence of ATP energy, thereby activating ubiquitin. b. Binding: The activated Ub is then transferred to ubiquitin-conjugating enzymes (E2s), binding to the Cys residue of E2s through a thioester bond. All E2s share a conserved core structural domain of approximately 150 amino acid residues, featuring a central Cys residue that dictates their enzymatic activity. This dynamic process involves the E2 shuttling between E1 and E3, as E1 and E3 are linked to the E2 by the same motif, forming an essential part of the reaction cycle. c. Ligations: Activated ubiquitin is either attached directly to the protein substrate via the E2 or is attached to the substrate by ubiquitin-protein ligases (E3). E3 transfer ubiquitin to the target protein through the formation of an amino-isopeptide bond between the carboxyl terminus of ubiquitin and the ε-amino group of the target protein’s Lys residue (9) (Figure 1). Among these, E3 plays a key role in the ubiquitination pathway by connecting E2 to the specific substrate, transferring the activated ubiquitin chain to the lysine residue of the specific substrate, and degrading the protein purposely by recognizing the multimeric ubiquitin chain. The specific recognition of various substrates by the complex and diverse E3 family members enables the ubiquitination pathway to demonstrate a high level of selectivity in protein degradation.

The process of attaching a single ubiquitin to an intracellular protein is known as monoubiquitination. When multiple Ub monomers simultaneously attach multiple different lysine residues in a target protein, it is referred to as multiubiquitination. Additionally, multiple ubiquitin monomers can form a polyubiquitinated chain on a target protein through different Lys residues (Lys6, Lys11, Lys27, Lys29, Lys33, Lys48, and Lys63), or Met1 residues can be interlinked to the target protein to create a polyubiquitinated chain (such as K48chain,K63chain), known as polyubiquitination (10) (Figure 1). The polyubiquitinated Ub residues determine the structure of the Ub chain. For example, the M1 chain and the K63 chain adopt an “open” conformation similar to a linear chain, while the K48 chain has a compact “forked” globular conformation (11). All these different ubiquitination structures and linkages and their combinations form a highly complex “ubiquitin code” that determines the future fate of target proteins (12). Overall, the functions of different forms of ubiquitination can be summarized in three aspects: a. Alter the stability of target proteins, causing them to be degraded by UPS; b. Alter the functional activity of target proteins; and c. Shift the localization of target proteins.

Most protein clearance in cells relies on ubiquitin, as proteins must be covalently modified with Ub before entering the proteasome for degradation. Proteins tagged with polyubiquitin can be identified by the proteasome. Research from Maniv et al. shows that dysregulation of ubiquitination may contribute to the development of AD (13). Ubiquitination modification is a dynamic and reversible process. E1/2/3 enzymes positively catalyze the ubiquitination reaction, while deubiquitinating enzymes (DUBs) reverse the process of ubiquitination. DUBs have been found to promote AD by cleaving ubiquitin from ubiquitination-modified substrate proteins, causing the substrate proteins to evade degradation by the proteasome (14). Research has found that ubiquitin levels were significantly elevated in the AD brain as measured by immunoassay. Ubiquitin-positive pathological protein aggregates in AD such as Aβ peptide, whose levels show a significant increase (15). Label-free mass spectrometry (MS)-based proteomic analysis has showed an 80% increase in ubiquitination levels in AD, with a total of 800 ubiquitination sites (15). Furthermore, the ubiquitination cascade has emerged as an attractive target for therapeutic intervention in AD (16).

## 3 Ubiquitination modification of AD-related proteins

### 3.1 Ubiquitination of Tau

Tau proteins are microtubule-associated proteins (MAPs) predominantly located in the axons of neurons within the central nervous system. Their primary functions include promoting and maintaining microtubule protein formation, stability, and regulating axonal transport (17). Anomalies in the aggregation of tau proteins closely correlate with neuronal loss and cognitive dysfunction. Excessive phosphorylation causes conformational changes in tau leading to tau protein aggregation, which is a toxic form of tau protein (18). Tau polymerization and degradation are regulated by a variety of post-translational modifications, among which ubiquitination modifications are critical for Tau to enter the degradation system. Phosphorylated Tau can be degraded by ubiquitination into the proteasome and lysosome (19, 20). The process of ubiquitination modification can be reversed by deubiquitinases (DUBs), including ubiquitin-specific protease 10 (USP10), a highly conserved deubiquitinating enzyme expressed widely in the brain, which promotes the aggregation of tau (21). In AD, the microtubule-associated protein tau has the highest number of ubiquitination sites per protein among ubiquitinated proteins (22), highlighting the essential role of the ubiquitination modification process carried out by tau via the Ubiquitin-Proteasome System (UPS) in AD.

**Figure 2:**
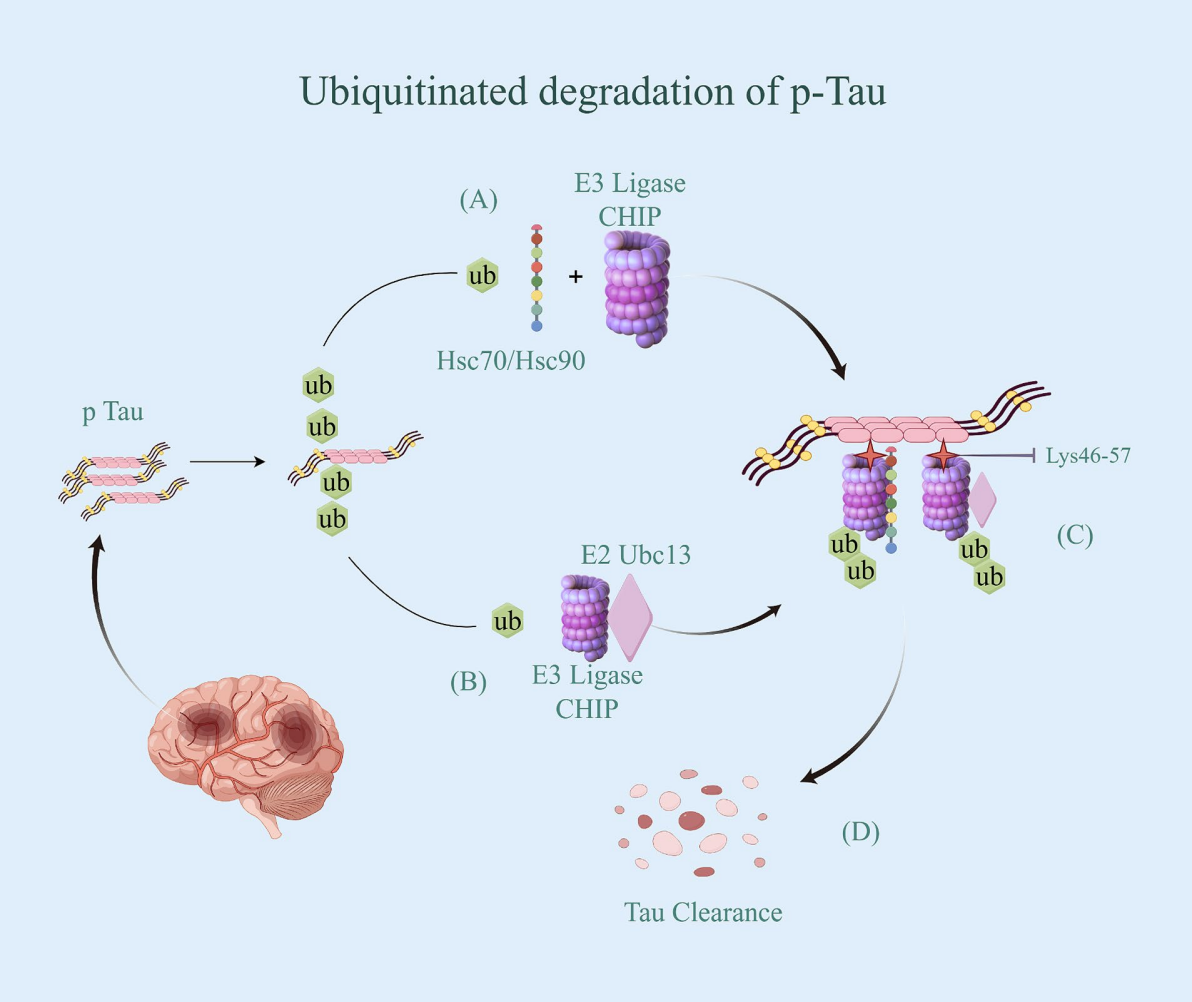
Ubiquitinated degradation of p-Tau. **(A)** CHIP binding to Hsc70/Hsp70 or Hsp90 chaperones transfer the activated Ub. **(B)** CHIP attach to the E2 enzyme Ubc13, and bond the activated Ub. **(C)** CHIP with Ub specifically bind to p-Tua’s Lysine residues 46–57 in N-terminal and C-terminal regions, and promote p-Tua’s ubiquitination *in vivo* and *in vitro*. **(D)** p-Tua with ubiquitin be identified by the proteasome, thereby being degraded.

The E3 CHIP plays a role in regulating the ubiquitin-dependent degradation of Tau *in vivo*. CHIP is part of the RING/U-box-type family, This family make up the largest E3 family that contains the RING or U-box catalytic domain (23), they do not bind ubiquitin directly but mediate the transfer of ubiquitin from bound E2 (E2-Ub) to the target substrate (24). CHIP acts as a quality regulator for the cellular proteome and is specifically designed to target misfolded proteins for degradation (25). On the one hand, CHIP can directly bind to Tau and promote its ubiquitination *in vivo* and *in vitro* (26). CHIP promotes the degradation of phosphorylated tau via UPS by binding to Hsc70/Hsp70 or Hsp90 chaperones (27). More precisely, the lysine clamp found in the CHIP tetrapeptide repeat sequence (TPR) structural domain, formed by the odd-numbered helices, attracts Hsp70 and Hsp90 family members by selectively binding to their C-terminal (I/M) EEVD motifs. This process completes the ubiquitination modification (28). On the other hand, CHIP has the capability to attach to the E2 enzyme Ubc13, which is responsible for catalyzing the ubiquitination of multiple lysine residues in the four repeat regions of Tau, even in the absence of Hsp70 (29). These lysine residues are largely present in Tau filaments from the brains of AD patients (30) (Figure 2). Tau protein serves as a substrate for CHIP by binding to it through various interaction sites, with a predominant presence in the N-terminal and C-terminal regions (specifically at residues 46–57 and 413–428). Additionally, there are some interactions in the region which is rich with proline and part of the N2 structural domain. The multisite recognition pattern is facilitated by the remarkable flexibility of Tau, as it is a long and intrinsically disordered polypeptide capable of exploring a large conformational space. This allows it to provide multiple individual contact points for interactions with protein chaperones (31).

CHIP, serving as a co-chaperone and an E3 ubiquitin ligase, is responsible for the ubiquitination and degradation of proteins. It plays a significant role in the development of AD (32). CHIP controls protein folding homeostasis that determines whether to refold or dephosphorylate pathologic aggregates in neural cells. If CHIP is not able to function properly, the degradation process will be severely compromised and accumulation of proteins will occur because of overburdened proteasomal and lysosomal systems (33). It has been reported that CHIP-positive tau inclusions were detected in AD (34). With the increase of research reports in recent years, it has been found that in addition to CHIP, E3 ubiquitin ligases such as Parkin (35), TRAF6 (36), and Hrd1 (37) also have the capacity to mediate the degradation of phosphorylated Tau and complete ubiquitination modification through the UPS pathway.

### 3.2 Ubiquitination of APP

APP is a type I single-channel transmembrane protein that undergoes processing and sorting by the endoplasmic reticulum and Golgi/trans-Golgi network before being secreted to the cytoplasmic membrane. Upon reaching the cytoplasmic membrane, it can be cleaved by α-secretase, leading to the production of the neuroprotective sAPPα fragment (38). Aβ acts as a product of a series of enzyme digestion pathways. Its over-production/aggregation is the main pathological feature of AD. The post-translational modification of APP plays a key regulatory role in the production and degradation of Aβ (39). Abnormalities in the ubiquitin modification process of APP have been identified as central to the pathological changes induced by Aβ deposition in AD (40).

The ubiquitin-dependent degradation of APP is primarily facilitated by the E3 ligases HRD1 and FBL2 through the UPS pathway (41, 42). HRD1 is an E3 ligase located in the endoplasmic reticulum that is upregulated during endoplasmic reticulum stress and is known to prevent endoplasmic reticulum stress-induced apoptosis (43). Study about loss of HRD1-mediated protein degradation have shown that the protein levels of HRD1 are significantly decreased in the cerebral cortex of Alzheimer’s disease patients, leading to the accumulation of APP (41). HRD1 acts as an E3 ligase for APP, binding specifically to APP at the proline-rich region of HRD1. This interaction facilitates the ubiquitination and subsequent proteasome-dependent degradation of APP, ultimately reducing the production of Aβ (41). However, the exact ubiquitin site of HRD1 binding to APP remains unclear, warranting further research to predict and validate this binding. F-box and leucine rich repeat protein2 (FBL2) belongs to the family of F-box proteins (44). It contains an F-box domain and also has 11 leucine-rich repeat regions for interaction with specific substrates. The expression of FBL2 which is the component of the complex E3 ubiquitin ligase Skp1-Cullin1-F-box (SCF) is reduced in the brains of AD patients (45). Binding FBL2 to APP and promoting its ubiquitination can reduce Aβ production. In this process, FBL2 facilitates the ubiquitination of both intracellular and cell surface APP. The ubiquitinated intracellular APP is then degraded by the proteasome, while the ubiquitinated cell surface APP does not undergo endocytosis. It subsequently reduces the amount of APP protein in lipid rafts and β-secretase APP cleavage (42). APP, as a substrate protein, binds specifically to FBL2 mainly through site lysine 651. What’s more, FBL2 can regulate APP metabolism by promoting ubiquitination at this site.

### 3.3 Ubiquitination of the Ferroptosis-related protein DMT1

Ferroptosis is an iron-dependent, novel mode of programmed cell death distinct from apoptosis, cell necrosis, and cell autophagy (46). The development of AD is closely linked to Ferroptosis (47), the iron accumulation has been shown to accelerate age-related plaque deposition and the production of neurogenic fiber tangles (48, 49). Abnormalities in proteins associated with iron uptake, storage, and export can cause an imbalance in iron homeostasis and induce cellular iron death. Ubiquitination is one of the most important posttranslational modifications for proteasomal degradation of target proteins mediated by specific ligases, and it is involved in Ferroptosis, and protein degradation by regulating protein stability (50). The transmembrane transport of iron ions is crucial for regulating cellular iron balance. Divalent metal transporter protein 1 (DMT1), a widely distributed transmembrane metal ion transporter, is responsible for the uptake of various divalent metal ions, including ferric ions (51). It was also found that DMT1 exhibits high expression levels in AD brains (52), and a pathological increase in DMT1 levels induces neurofibrillary tangles (53). DMT1 has 12 transmembrane segments and is expressed in neurons, allowing for the doping of metals from the extracellular environment and/or recycling of endosomes (54). Once Fe^2+^ are translocated into the cytoplasm via DMT1, they are metabolically utilized to maintain iron homeostasis. The degradation of DMT1 is primarily regulated by ubiquitination modifications, and this process is crucial for preventing iron accumulation and neuronal apoptosis (55). Therefore, the ubiquitinated degradation of DMT1 is also significant in the context of AD.

**Figure 3:**
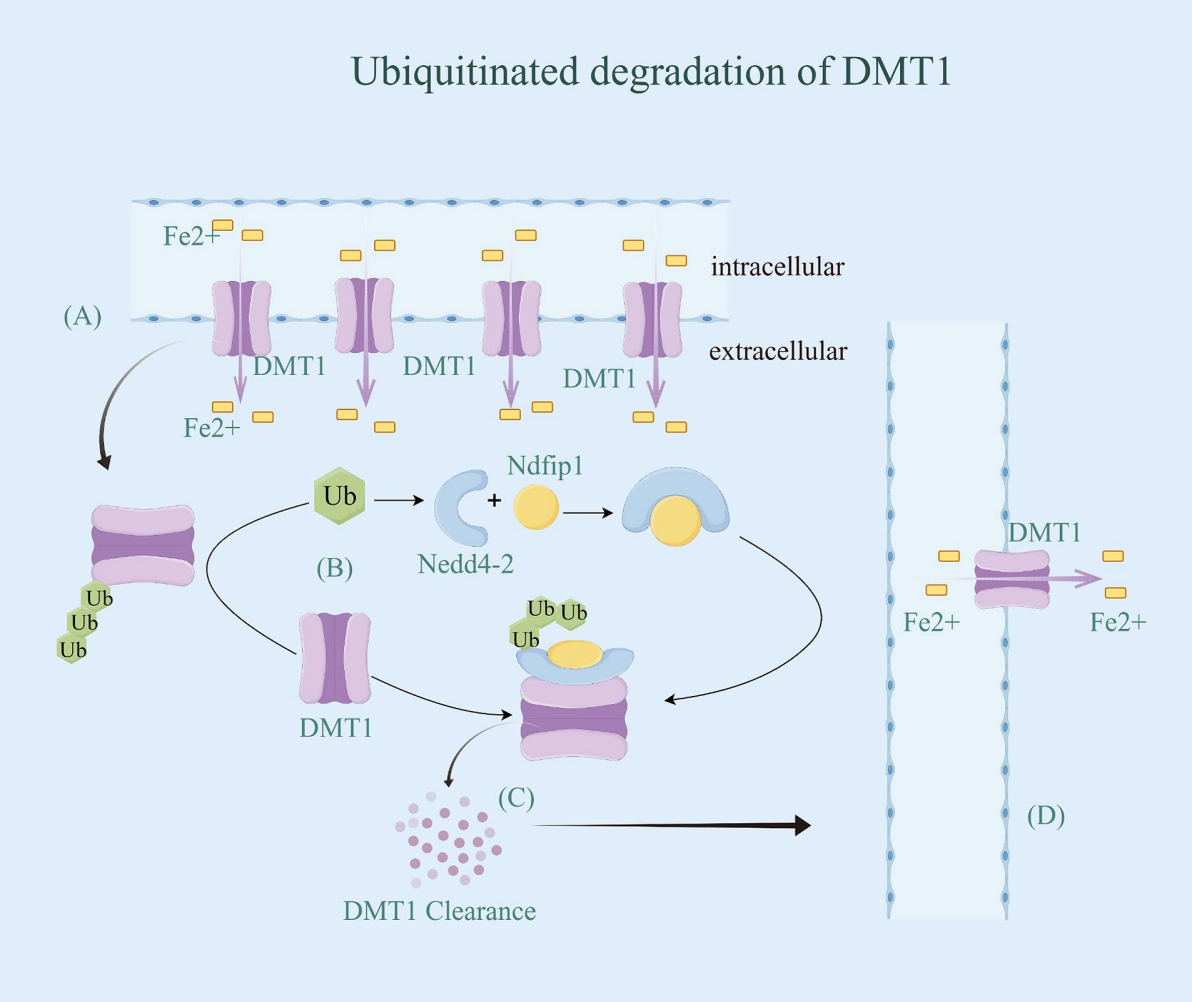
Ubiquitinated degradation of DMTl. **(A)** DMT1 has 12 transmembrane segments and is expressed in neurons, allowing for the doping of Fe^2+^ from the extracellular environment and/or recycling of endosomes. Once Fe^2+^ are translocated into the cytoplasm via DMT1, they are metabolically utilized to maintain iron homeostasis. **(B)** Ndfip1 serves as a bridging protein that facilitates the recruitment of Nedd4-2 with Ub and binds to DMT1 to complete the ubiquitination and subsequent degradation of DMT1. **(C)** DMT1 with ubiquitin be identified by the proteasome, thereby being degraded. **(D)** With the downregulation of DMT1 expression and activity, intracellular iron accumulation be reduced.

The regulation of iron ion channels and transport proteins involves ubiquitination, mediated by the Nedd4 family of E3 ubiquitin ligases. These ligases typically interact with their substrates through WW structural domains, binding to specific motifs in the target proteins (56). However, not all potential targets of these E3 ligases contain these binding motifs. As a result, auxiliary proteins may facilitate the interaction between the Nedd4 family members and their targets (57). For instance, Nedd4-2 is a ubiquitin ligase for DMT1 polyubiquitination under metal-induced stress (54). Additionally, its interacting protein Ndfip1, also known as Nedd4WW structural domain binding protein 5 (N4WBP5) (58), has the ability to facilitate the ubiquitinated degradation of various target proteins, including DMT1 (59). Ndfip1 serves as a bridging protein that facilitates the recruitment of Nedd4-2 and binds to DMT1 to complete the ubiquitination and subsequent degradation of DMT1. It has been observed that reduced levels of Ndfip1 in the AD brain are linked to elevated DMT1 levels (60). Throughout the ubiquitination modification process, Ndfip1 plays a crucial role in targeting E3 ligase ubiquitinated proteins as an auxiliary articulating protein, forming a complex by specifically binding Nedd4-2 and DMT1 (54). This complex accomplishes the ubiquitination degradation of DMT1, resulting in the downregulation of DMT1 expression and activity, ultimately reducing intracellular iron accumulation (Figure 3). However, the precise ubiquitin site of Ndfip1/Nedd4-2-mediated DMT1 degradation remains unknown, and future studies are needed to predict and verify this ubiquitin site through experimental design.

### 3.4 Ubiquitination of AMPARs

Synapses are central for information transfer between neurons. Synaptic plasticity, the biological foundation of learning and memory, governs the structure and function of synapses (61). Structural plasticity is marked by changes in synapse number and morphology, while functional plasticity balances Long-term potentiation (LTP) and Long-term depression (LTD) activities (62). Impairment of synaptic functional plasticity correlates strongly with memory impairment in early AD (63, 64). AD is characterized by progressive and irreversible memory impairment, and associated with inhibition of LTP and enhancement of LTD in the hippocampus (65, 66). The establishment of memory and cognitive abilities is closely linked to synaptic plasticity. Impaired synaptic plasticity has been identified as one of the key causative factors and early pathological features of Alzheimer’s disease (AD) (67). It has been shown that synaptic adaptation and stability are ultimately regulated by synaptic proteins, including removal, addition, and post-translational modifications (68). Ubiquitin-dependent protein degradation is a critical step in memory consolidation (69). In recent years, a role for protein degradation by the ubiquitin–proteasome pathway in synaptic plasticity has been discovered (70, 71). Dong et al. reported that the proteasome plays a facilitatory role in the maintenance part of L-LTP (72). Their data indicate Proteasome inhibition leads to postsynaptic changes such as stabilization of newly synthesized proteins in dendrites, and causes presynaptic changes in the hippocampus such as modulation of transmitter release. These all show that UPS mediated degradation may have broad implications for synaptic plasticity under physiological conditions, as well as synaptic dysfunction in AD with which abnormal protein degradation is associated (73). Post-synaptic density AMPA receptors (AMPARs) play a crucial role in supporting synaptic transmission and plasticity (74). Increasing the number of AMPARs has been shown to enhance synaptic transmission efficiency (75). Impaired AMPAR function is associated with cognitive deficits in the early stages of AD, and its excitatory damage can lead to neurodegeneration in the late stages of the disease (76). Furthermore, post-translational ubiquitination of AMPAR has emerged as a significant factor in AD, regulating the surface expression of these receptors and playing a key role in the disease.

**Figure 4:**
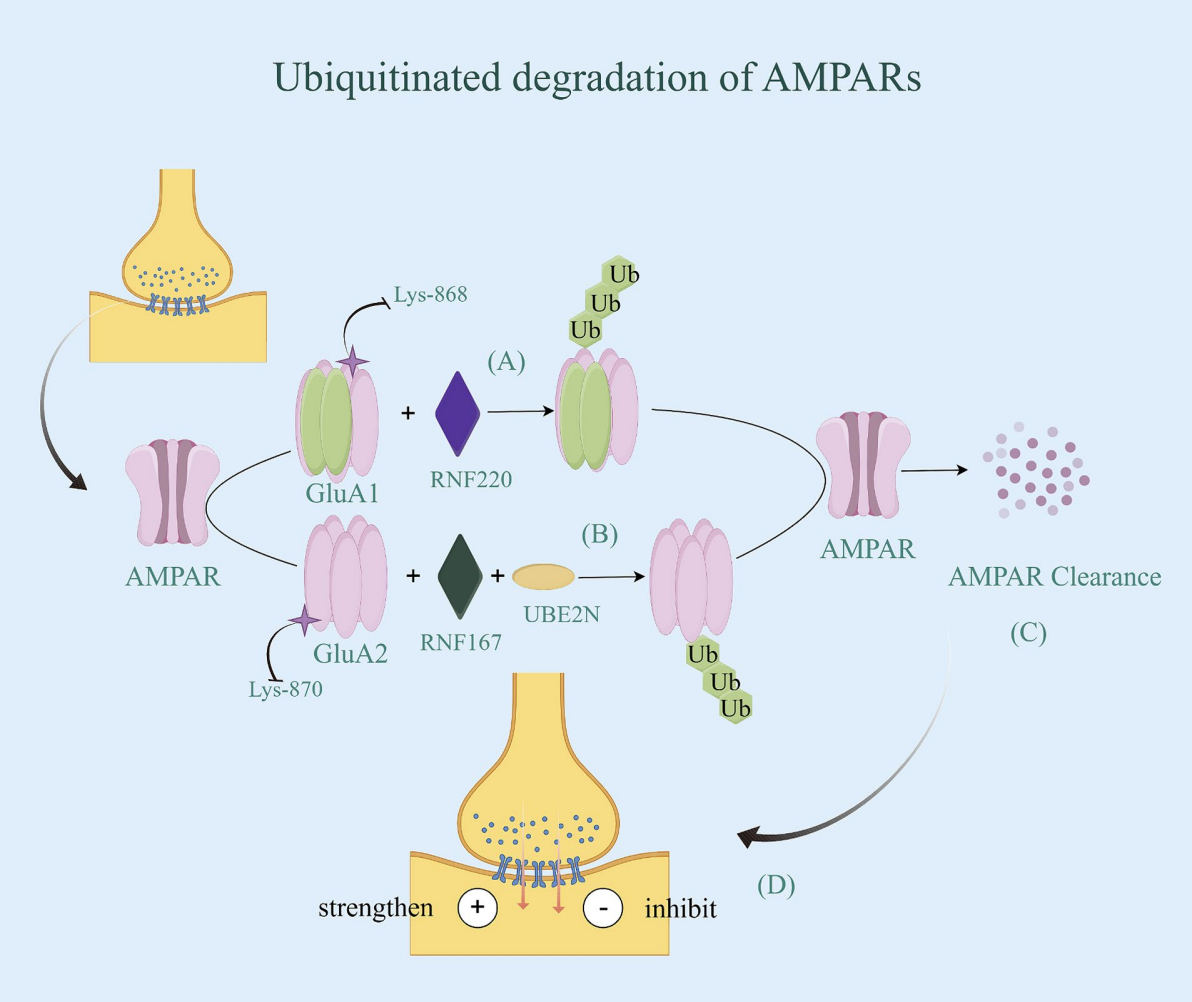
Ubiquitinated degradation of AMPARs. **(A)** The activity-dependent ubiquitination of GluA1 occurs at the C-terminal Lys-868 residue and is primarily mediated by the E3 RNF220. Their co-localization and binding to AMPARs trigger GluA1 ubiquitination and promote the internalized degradation of AMPARs. **(B)** GluA2, as one of the substrates of RNF167, is ubiquitinated by first binding to the E2 coupling enzyme UBE2N, and then the RING structural domain of RNF167 binds to the coupling enzyme between them, completing the ubiquitin degradation of GluA2, and enhancing synaptic transmission efficacy. Additionally, the ubiquitination site is mainly located on the Lys-870/Lys-882 residues at the C-terminal end of the GluA2 subunit. **(C)** AMPARs with ubiquitin be identified by the proteasome, thereby being degraded. **(D)** Due to the different physiological functions of the different subunits in the transport of AMPARs, ubiquitination of the GluA1 subunit inhibits synaptic transmission, whereas ubiquitination of the GluA2 subunit enhances synaptic transmission.

AMPARs, the primary mediators of excitatory synaptic transmission in the brain, consist of two dimers of a combination of four subunits (GluA1-GluA4). Additionally, different subunits are capable of conferring specific physiological properties to the AMPARs channel functions (77). Each subunit has a variable C-terminus, which is a key factor in the transport of AMPARs. Among them, the GluA1 and GluA2 subunits play important roles in cytosis and internalization (78). Ubiquitination of both subunits is critical for regulating the surface and synaptic expression of AMPARs (79). It has been shown that promoting GluA1 expression improves synaptic transmission efficacy, and its hyperubiquitination leads to amyloid-β (Aβ)-induced downregulation of surface AMPAR expression and inhibition of excitatory synaptic transmission (80). The activity-dependent ubiquitination of GluA1 occurs at the C-terminal Lys-868 residue and is primarily mediated by the E3 ligases Nedd4-1, Nedd4-2, and RNF220 (81–83). Their co-localization and binding to AMPARs trigger GluA1 ubiquitination and promote the internalized degradation of AMPARs. GluA2 is also an important subunit that determines the function of AMPAR, and its expression in tetramers correlates with the rate of inactivation, single-channel conductance, and Ca^2+^ permeability of AMPARs (84). The process of GluA2 internalization, where it is taken back into the synapse from the postsynaptic membrane, weakens synaptic transmission when increased (75). Consequently, an overactivation of receptors for AMPARs containing the GluA2 subunit results in dysfunction and potential death of neurons in AD. The RING-type E3 ubiquitin ligase, RNF167, is a transmembrane protein that resides in endosomes and lysosomes and is involved in regulating the endolysosomal pathway. GluA2, as one of the substrates of RNF167, is ubiquitinated by first binding to the E2 coupling enzyme UBE2N, and then the RING structural domain of RNF167 binds to the coupling enzyme between them, completing the ubiquitin degradation of GluA2 (85), and enhancing synaptic transmission efficacy. Additionally, the ubiquitination site is mainly located on the Lys-870/Lys-882 residues at the C-terminal end of the GluA2 subunit (79) (Figure 4). Notably, ubiquitination of the GluA1 subunit inhibits synaptic transmission, whereas ubiquitination of the GluA2 subunit enhances synaptic transmission. This is due to the different physiological functions of the different subunits in the transport of AMPARs.

## 4 Conclusion and discussion

Ubiquitin-dependent protein degradation is essential for neuronal health, where protein remodeling underlies specific brain processes such as memory and learning as well as synaptic plasticity (86). This paper not only explores the process of ubiquitination modification but also provides an overview of the ubiquitin degradation of proteins associated with Alzheimer’s disease (AD), particularly focusing on the significant role of E3 ubiquitin ligases in this process (Table 1). Considering the complexity and diversity of ubiquitination modifications, the discussion here is limited to the regulation of protein degradation. In fact, the signaling pathways involved in ubiquitination also play a crucial role. For instance, in the NF-κB pathway, the IκBα protein is degraded through ubiquitination modification, lifting the inhibition of NF-κB and enabling it to enter the nucleus to carry out transcriptional activities, regulating inflammatory and immune responses (87). Additionally, more E3 ubiquitin ligases have been implicated in AD, such as mitochondrial ubiquitin ligase (MITOL/MARCH5), situated in the outer membrane of mitochondria, and contributing to the maintenance of mitochondrial homeostasis by clearing protein aggregates that have accumulated on mitochondria (88). MITOL deficiency has been shown to disrupt mitochondrial dynamics, leading to mitochondrial damage and worsening cognitive decline in the APP/PS1 mouse model. However, further investigation is required to understand the detailed pathways of action and ubiquitin sites.

**Table 1:** Pathological mechanism of E3 involved in ubiquitination of AD-related proteins.

E3	Target protein	Ubiquitin site	Pathological mechanism
CHIP	Tua	Lys46-57/ Lys413-428	Catalyzes the ubiquitination of multiple lysine residues in the four repeat regions of Tau and promotes the degradation of p-Tau
HRD1	APP	Unknown	Binds specifically to APP at the proline-rich region of HRD1 and facilitates the ubiquitination degradation of APP, ultimately reducing the production of Aβ
FBL2	APP	Lys651	Binds to APP and promotes the ubiquitination of both intracellular and cell surface APP, ultimately reducing the production of Aβ
Nedd4-2	DMT1	Unknown	Binds specifically to DMT1 via auxiliary protein Ndfip1 and promotes ubiquitination degradation of DMT1, resulting in the downregulation of DMT1 expression and activity, ultimately reducing intracellular iron accumulation
RNF220	AMPARs-GluA1	Lys868	Binds to AMPARs trigger GluA1 ubiquitination and promotes the internalized degradation of AMPARs, ultimately inhibition of excitatory synaptic transmission
RNF167	AMPARs-GluA2	Lys870/ Lys882	Binds to the E2 coupling enzyme UBE2N, and then the RING structural domain of RNF167 binds to the coupling enzyme between them, completing the ubiquitin degradation of GluA2, ultimately enhancing synaptic transmission efficacy

AD is characterized as a protein-related disorder, with disturbances in protein homeostasis playing a crucial role. Compared to other neurodegenerative diseases such as Parkinson’s, which relies primarily on ubiquitin-dependent mitochondrial autophagy to accomplish protein degradation due to its defective mitochondrial function (89), AD relies primarily on UPS. UPS is an important post-translational modification for protein degradation and control of homeostasis. The enzymes involved in UPS, such as E1, E2, E3 ligases, and DUB, regulate disease-induced protein aggregation and degradation by controlling the degree of ubiquitination (90). During the ubiquitination degradation process of AD-associated proteins, E3, acting as a bridge to transfer activated Ub from E2 to the substrate proteins, is specifically recognized by the proteasome for degradation, which in turn delays the development of AD. On the other hand, DUBs remove Ub from substrate proteins by cleaving the peptide or isopeptide bond between Ub and substrate, rescuing the substrate proteins from the ubiquitination degradation pathway and preventing them from being degraded. This in turn leads to the accumulation of AD-associated neurotoxicity proteins and facilitates the development of AD. Extensive research has been dedicated to identifying novel targets for AD treatment. Given the complex mechanisms and diverse proteins involved, this paper highlights the significance of four major protein degradation pathways in AD pathogenesis, emphasizing the potential of targeting key components in these pathways for therapeutic interventions. With the increase in clinical studies, modulators based on ubiquitination-targeted therapy for AD have emerged. For instance, the modulator geniposide increases HRD1 expression, promoting the phosphorylation of inositol requiring enzyme 1 alpha (IRE1α), resulting in accelerated degradation of amyloid precursor protein (APP) (91). Another modulator, X-box binding protein (XBP-1), indirectly decreases the expression and activity of β-site APP cleaving enzyme (BACE1) by up-regulating HRD1, reducing amyloid-beta (Aβ) production (92). The small molecule agonists Sulforaphane, Anisomycin and Peptidoglycan (PGN), which promote CHIP expression, inhibit the progression of AD by promoting the expression of CHIP in the cerebral cortex and hippocampus, leading to ubiquitinated degradation of phosphorylated Tau proteins, and may be targeted drugs for the treatment of neurological disorders (93). Additionally, the involvement of deubiquitinating enzymes (DUBs), including ubiquitin-specific protease14 (USP14), in AD development has been identified. USP14, a proteasome-associated ubiquitin-specific protease, plays a critical role in neurodegenerative diseases, inflammatory responses, tumorigenesis and other aspects (94). Studies have shown that USP14 inhibits ubiquitination of hyperphosphorylated Tau proteins and induces further exacerbation of AD. The small molecule inhibitor of USP14, IU1-47, has been shown to be targetable for the treatment of AD by accelerating the degradation of hyperphosphorylated Tau proteins through inhibition of USP14 activity (95). Lee et al. conducted preclinical study to test whether the small molecule inhibitor of USP14, IU1, could inhibit the trimming of ubiquitin chains by the proteasome and whether IU1 could enhance proteasome function in cells (96). The result shows IU1 enhanced proteasome function and was helpful for Tua degradation. Furthermore, it could potentially be used to eliminate misfolded proteins more effectively. However, the efficacy of the IU1 series of compounds for the treatment of AD by targeting USP14 requires further clinical trials studies.

Although ubiquitination events are considered a promising target for AD therapy, the identification of highly potent and specific E3 and DUB modulators has not been successful due to the complexity of the enzyme cascade involved in ubiquitin-binding. E3 and DUB have a synergistic or exclusive role in functionality. Therefore, more in-depth research is needed to explore the mechanism of E3 and DUB action in AD, as well as the balance between ubiquitination and deubiquitination. In recent years, the treatment of AD by proteolysistargeting chimera (PROTAC) is also very promising. However, there are still few ligands for E3 ligase, which has a certain degree of limitation on the treatment of AD. Whether there are new E3s and related target proteins or known E3s and target proteins that can regulate the development of AD through novel mechanisms needs future studies. Furthermore, whether they can be used clinically as therapeutic targets for AD also requires in-depth study with the aim of providing new approaches for the future treatment of AD.
